# Expanded Recommendations for Use of Pneumococcal Conjugate Vaccines Among Adults Aged ≥50 Years: Recommendations of the Advisory Committee on Immunization Practices — United States, 2024

**DOI:** 10.15585/mmwr.mm7401a1

**Published:** 2025-01-09

**Authors:** Miwako Kobayashi, Andrew J. Leidner, Ryan Gierke, Wei Xing, Emma Accorsi, Pedro Moro, Mini Kamboj, George A. Kuchel, Robert Schechter, Jamie Loehr, Adam L. Cohen

**Affiliations:** ^1^Division of Bacterial Diseases, National Center for Immunization and Respiratory Diseases, CDC; ^2^Immunization Services Division, National Center for Immunization and Respiratory Diseases, CDC; ^3^Immunization Safety Office, CDC; ^4^Memorial Sloan Kettering Cancer Center, Joan and Sanford Weill Medical College of Cornell University, New York, New York; ^5^UConn Health, Farmington, Connecticut; ^6^California Department of Public Health; ^7^Cayuga Family Medicine, Ithaca, New York.

**Summary**

**What is already known about this topic?**

Before October 2024, a single dose of 15-valent, 20-valent, or 21-valent pneumococcal conjugate vaccine (PCV), was recommended for adults aged 19–64 years with risk conditions for pneumococcal disease and for all adults aged ≥65 years.

**What is added by this report?**

On October 23, 2024, the Advisory Committee on Immunization Practices recommended a single dose of PCV for all adults aged ≥50 years who are PCV-naïve or who have unknown vaccination history. The risk-based recommendation for adults aged 19–49 years is unchanged.

**What are the implications for public health practice?**

The updated, expanded age-based recommendation is expected to improve pneumococcal disease prevention in adults aged 50–64 years, particularly among demographic groups experiencing higher disease rates.

## Abstract

Before October 2024, the Advisory Committee on Immunization Practices (ACIP) recommended use of a pneumococcal conjugate vaccine (PCV) for all adults aged ≥65 years, as well as for those aged 19–64 years with risk conditions for pneumococcal disease who have not received a PCV or whose vaccination history is unknown. Options included either 20-valent PCV (PCV20; Prevnar20; Wyeth Pharmaceuticals) or 21-valent PCV (PCV21; CAPVAXIVE; Merck Sharp & Dohme) alone or 15-valent PCV (PCV15; VAXNEUVANCE; Merck Sharp & Dohme) in series with 23-valent pneumococcal polysaccharide vaccine (PPSV23; Pneumovax23; Merck Sharp & Dohme). There are additional recommendations for use of PCV20 or PCV21 for adults who started their pneumococcal vaccination series with 13-valent PCV (PCV13; Prevnar13; Wyeth Pharmaceuticals). The ACIP Pneumococcal Vaccines Work Group employed the Evidence to Recommendations framework to guide its deliberations on expanding the age-based PCV recommendation to include adults aged 50–64 years. On October 23, 2024, ACIP recommended a single dose of PCV for all PCV-naïve adults aged ≥50 years. Recommendations for PCVs among adults aged 19–49 years with risk conditions and PCV13-vaccinated adults have not changed from previous recommendations. This report summarizes evidence considered for these recommendations and provides updated clinical guidance for use of PCV.

## Introduction

*Streptococcus pneumoniae* (pneumococcus) is a common bacterial cause of respiratory tract infections, bacteremia, and meningitis. Widespread use of pneumococcal conjugate vaccine (PCV) in children reduced the incidence of pneumococcal disease, both among children through direct effects and among older children and adults who have not received PCV through indirect effects (i.e., reduction in disease incidence in the population because of decreased transmission of pneumococcus from children) (*1**,**2*). However, persons with underlying conditions or factors that increase their risk for pneumococcal disease (risk conditions)* and older adults experience higher pneumococcal disease rates. In addition, racial disparities in pneumococcal disease incidence persist, including higher rates among non-Hispanic Black or African American (Black) and non-Hispanic American Indian or Alaska Native (AI/AN) adults (*3*).

Before its October meeting, the Advisory Committee on Immunization Practices (ACIP) recommended receipt of a single dose of PCV for all adults aged ≥65 years and those aged 19–64 years with a risk condition who have not received PCV or whose vaccination history is unknown. Options included either 20-valent PCV (PCV20; Prevnar20; Wyeth Pharmaceuticals) (*4*) or 21-valent PCV (PCV21; CAPVAXIVE; Merck Sharp & Dohme) (*5*) alone, or 15-valent PCV (PCV15; VAXNEUVANCE; Merck Sharp & Dohme) (*6*) followed by 23-valent pneumococcal polysaccharide vaccine (PPSV23; Pneumovax23, Merck Sharp & Dohme) (*7*). Additional recommendations are applicable for use of PCV20 or PCV21 for adults who commenced their pneumococcal vaccination series with 13-valent PCV (PCV13; Prevnar13, Wyeth Pharmaceuticals) (*8**,**9*).

In June 2024, ACIP recommended PCV21 as an option for adults who are recommended to receive PCV and proposed a review of available evidence to determine whether data supported lowering the age-based recommendation to ≥50 years for all recommended PCVs (*8*). The approval of PCV21, which was specifically developed to target pneumococcal serotypes that commonly cause disease in adults (Figure), was seen as a unique opportunity to reduce pneumococcal disease incidence and health disparities among U.S. adults. This report summarizes the evidence considered by ACIP regarding the expansion of the age-based recommendation to include adults aged 50–64 years, highlighting considerations of pneumococcal disease incidence and mortality, health disparities, and resource use.

**FIGURE:**
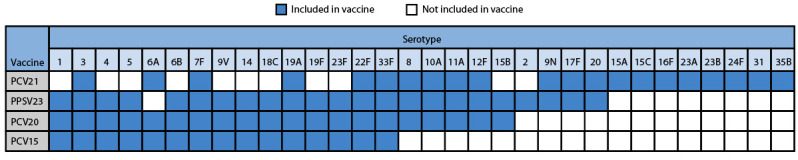
Serotypes*^,^^†^ included in pneumococcal vaccines currently recommended for adults — United States, 2024 **Abbreviations:** PCV = pneumococcal conjugate vaccine; PCV15 = 15-valent PCV; PCV20 = 20-valent PCV; PCV21 = 21-valent PCV; PPSV23 = 23-valent pneumococcal polysaccharide vaccine. * PCV21 is approved for the prevention of invasive pneumococcal disease caused by serotype 15B based upon prespecified criteria for the proportion of participants with fourfold or more rise in opsonophagocytic activity responses. https://www.fda.gov/media/179426/download?attachment ^†^ PCV21 contains serotype 20A.

## Methods

During July–October 2024, the ACIP Pneumococcal Vaccines Work Group considered PCV use among PCV-naïve adults aged 50–64 years within the Evidence to Recommendations (EtR) framework.^†^ Published and unpublished data on pneumococcal disease incidence and mortality, pneumococcal vaccination coverage, and economic models of age-based PCV use at age ≥50 years were reviewed; and findings were summarized by race and ethnicity whenever available (*3**,**10*). Previous Grading of Recommendations, Assessment, Development and Evaluation (GRADE) reviews for PCV15, PCV20, and PCV21 (*8**,**11**,**12*) were supplemented by an updated search of MEDLINE, (using PubMed) and ClinicalTrials.gov to identify additional literature on safety and immunogenicity. Postlicensure safety data on PCV20 from the Vaccine Adverse Event Reporting System (VAERS) and an analysis using Centers for Medicare & Medicaid Services (CMS) data were reviewed.

## Rationale and Evidence

### Pneumococcal Disease Incidence in Adults Aged ≥19 Years

Pneumococcal pneumonia, accounting for 12%–13% of all hospitalized pneumonia cases, has been estimated to result in approximately 225,000 U.S. adult hospitalizations annually (*13**–**15*). Among adults aged 50–64 years with invasive pneumococcal disease (IPD) and those hospitalized with pneumococcal pneumonia, approximately 90% had one or more risk condition (*3**,**14*). Before the COVID-19 pandemic, approximately 30,000 IPD^§^ cases occurred annually among U.S. adults (*16*). In 2022, adults aged 50–64 years experienced IPD incidence and mortality rates of 13.2 and 1.8 per 100,000 population, respectively. These rates were higher than those in all other age groups except adults aged ≥65 years, whose incidence and mortality rates were 17.2 and 2.7 per 100,000 population, respectively (*1*). According to CDC’s Active Bacterial Core surveillance (ABCs) data, during 2018–2022 (before PCV20 was widely used and before PCV21 approval among adults), 56% and 83%^¶^ of IPD cases were due to pneumococcal serotypes contained in PCV20 and PCV21 in adults aged 50–64 years, respectively (*17*).

### Racial Disparities in Pneumococcal Disease Incidence and Vaccination Coverage

An estimated 32%–54% of adults aged 50–64 years had at least one risk condition that qualifies for risk-based pneumococcal vaccination.** However, 2022 Behavioral Risk Factor Surveillance System data showed that only 37% of adults aged 50–64 years with a risk-based vaccination recommendation received a pneumococcal vaccine, compared with 70% of adults aged ≥65 years with an age-based recommendation; racial disparities in vaccination rates were apparent^††^ (*3*). ABCs data showed that IPD rates among Black adults peaked at a younger age (55–59 years) compared with rates among non-Black adults whose IPD rates increased with increasing age (*3*). Although PCV13 use among U.S. children has reduced disparities in PCV13-type IPD incidence in adults, likely because of indirect effects; remaining racial disparities are driven by non-PCV13 serotypes, with non-PCV13 serotype IPD rates among AI/AN and Black adults (25 and 10 per 100,000 population, respectively) exceeding the population average of six per 100,000 (*3*).

### PCV Immunogenicity and Safety from Clinical Trials

An updated literature search identified six PCV15 trials (*18**–**23*), three PCV20 trials (*24**–**26*), and seven PCV21 trials (*27**–**32*) that included immunogenicity and safety data for adults aged ≥50 years. Summary of evidence from the updated literature search remained essentially unchanged from previous summaries (*3**,**8**,**11**,**12*). Compared with PCV13, PCV15 met noninferiority criteria for all shared PCV13 serotypes, and immune responses for non-PCV13 serotypes 22F and 33F were statistically significantly higher. PCV20 met noninferiority criteria for all PCV13 serotypes compared with PCV13 and for six of seven non-PCV13 serotypes (not met for serotype 8) compared with PPSV23 (*24**–**26*). Compared with PCV20, PCV21 met noninferiority criteria for 10 of 10 shared serotypes, and immune responses for 10 of 11 unique serotypes were statistically significantly higher (not met for serotype 15C). No vaccine-related serious adverse events (SAEs) were reported after PCV15 or PCV20 administration; two vaccine-related SAEs had been previously reported after PCV21 administration (*8*).

### PCV20 Postlicensure Safety Data

Analysis of reports to VAERS after PCV20 administration in adults aged ≥19 years during October 2021–August 2024 showed a signal for Guillain-Barré syndrome (GBS); however, the overall reporting rate remained low (0.7 cases per million doses distributed) (*3*). Primary analysis of CMS data through May 2024 showed a statistically significant signal for GBS^§§^ after PCV20 administration in Medicare beneficiaries aged ≥65 years. However, the signal was not statistically significant when applying an alternative GBS definition in sensitivity analysis or adjusted for the positive predictive value of diagnostic codes compared with confirmation by chart review (*3*).

### Economic Analysis

Two economic models (Tulane-CDC and Merck) assessed the cost-effectiveness of PCV20 and PCV21 use among PCV-naïve adults aged 50–64 years (*10*). A third model (Pfizer) assessed the cost-effectiveness of PCV20 use only (*10*). All three models used quality-adjusted life-year (QALY) as the primary health outcome. The Tulane-CDC model estimated costs of $131,023–$214,430 per QALY gained for PCV21 and $251,037–$546,811 for PCV20. The Merck model estimated $251,048–$425,455 per QALY gained for PCV21 and $548,114–$879,117 for PCV20. The Pfizer model estimated $56,376–$133,524 per QALY gained for PCV20. Cost-effectiveness estimates were most sensitive to assumptions about indirect effects from pediatric vaccination and duration of protection from vaccination. Limitations of the models included uncertainties about duration of protection from vaccination, magnitude of indirect effects from pediatric vaccination, and impact of future supplementary pneumococcal vaccine doses for adults.

## Recommendations for Use of PCV

ACIP recommended PCV for all PCV-naïve adults aged ≥50 years. Recommendations for PCVs for adults aged 19–49 years with a risk condition and for adults who have previously received PCV13 remain unchanged (Table) (*8*). The recommendation was supported by several factors, including the potential to improve vaccination coverage and reduce pneumococcal disease incidence and mortality in adults aged 50–64 years, particularly among demographic groups experiencing higher disease rates. Ease of implementing consistent age-based recommendations for all PCVs was also considered. Uncertainties regarding key assumptions guiding the economic models and higher cost per QALY estimates for PCV20 compared with PCV21 were acknowledged.

**TABLE:** Clinical guidance for implementing pneumococcal vaccine recommendations for adults aged ≥19 years — United States, October 2024

Risk or age group	Vaccine received previously	Options for vaccination
Adults aged ≥50 years	None or PCV7 only at any age	A single dose of PCV21, PCV20, or PCV15. If PCV15 is administered, a single dose of PPSV23* should be administered ≥1 year after the PCV15 dose. A minimum interval of 8 weeks can be considered if PCV15 is used in adults with an immunocompromising condition,^†^ cochlear implant, or CSF leak.
	PPSV23 only	A single dose of PCV21, PCV20, or PCV15 ≥1 year after the last PPSV23 dose.
	PCV13 only	A single dose of PCV21 or PCV20 ≥1 year after the PCV13 dose.
	PCV13 at any age and PPSV23 at age <65 years	A single dose of PCV21 or PCV20 ≥5 years after the last pneumococcal vaccine dose.
	PCV13 at any age and PPSV23 at age ≥65 years	Shared clinical decision-making is recommended regarding administration of either a single dose of PCV21 or PCV20 for any adult aged ≥65 years who has completed the recommended vaccination series with both PCV13 and PPSV23 (i.e., PPSV23 administered at age ≥65 years) but PCV21, PCV20, or PCV15 not yet received. If a decision to administer PCV21 or PCV20 is made, a single dose is recommended ≥5 years after the last pneumococcal vaccine dose.
Adults aged 19–49 years with an immunocompromising condition,^†^ a CSF leak, or a cochlear implant	None or PCV7 only at any age	A single dose of PCV21, PCV20, or PCV15. If PCV15 is used, administer a single dose of PPSV23* ≥8 weeks after the PCV15 dose.
	PPSV23 only	A single dose of PCV21, PCV20, or PCV15 ≥1 year after the last PPSV23 dose.
	PCV13 only	A single dose of PCV21 or PCV20 administered ≥1 year after the PCV13 dose.
	PCV13 and 1 dose of PPSV23	A single dose of PCV21 or PCV20 ≥5 years after the last pneumococcal vaccine dose. The pneumococcal vaccination series is complete, and it need not be followed by additional pneumococcal vaccine doses.
	PCV13 and 2 doses of PPSV23	The pneumococcal vaccination recommendations should be reviewed again when the person turns age 50 years. Alternatively, a single dose of either PCV21 or PCV20 should be administered ≥5 years after the last pneumococcal vaccine dose. If PCV21 or PCV20 is used, the series is complete, and it need not be followed by additional pneumococcal vaccine doses.
Adults aged 19–49 years with chronic medical conditions^§^	None or PCV7 only at any age	A single dose of PCV21, PCV20, or PCV15. If PCV15 is administered, a single dose of PPSV23* should be administered ≥1 year after the PCV15 dose.
	PPSV23 only	A single dose of PCV21, PCV20, or PCV15 ≥1 year after the last PPSV23 dose.
	PCV13 only	A single dose of PCV21 or PCV20 ≥1 year after the PCV13 dose.
	PCV13 and 1 dose of PPSV23	The pneumococcal vaccination recommendations should be reviewed again when the person reaches age 50 years.

**Abbreviations**: CSF = cerebrospinal fluid; PCV = pneumococcal conjugate vaccine; PCV7 = 7-valent PCV; PCV13 = 13-valent PCV; PCV15 = 15-valent PCV; PCV20 = 20-valent PCV; PCV21 = 21-valent PCV; PPSV23 = 23-valent pneumococcal polysaccharide vaccine.

* For adults who have received PCV15 but have not completed their recommended pneumococcal vaccine series with PPSV23, 1 dose of PCV21 or PCV20 may be used if PPSV23 is not available.

^†^ Chronic renal failure, nephrotic syndrome, immunodeficiency, iatrogenic immunosuppression, generalized malignancy, HIV infection, Hodgkin disease, leukemia, lymphoma, multiple myeloma, solid organ transplant, congenital or acquired asplenia, or sickle cell disease or other hemoglobinopathies.

^§^ Alcoholism; chronic heart disease, including congestive heart failure and cardiomyopathies; chronic liver disease; chronic lung disease, including chronic obstructive pulmonary disease, emphysema, and asthma; cigarette smoking; or diabetes mellitus.

### Selection of PCV in Populations with High Proportions of Serotype 4 Pneumococcal Disease

In many U.S. settings, PCV21 is expected to cover more circulating pneumococcal strains than do other recommended PCVs. In certain populations in which ≥30% of pneumococcal disease^¶¶^ is due to serotype 4, pneumococcal vaccines that include serotype 4 (PCV20 alone or PCV15 and PPSV23 in series) (Figure) are expected to provide broader serotype coverage against locally circulating strains than does PCV21 (Box).

BOX **Clinical guidance on selection of pneumococcal conjugate vaccine in communities with high percentages of serotype 4 pneumococcal disease — United States, 2024**

- PCV21 contains eight pneumococcal serotypes that are not included in previously recommended pneumococcal vaccines (i.e., PCV15, PCV20, and PPSV23). However, PCV21 does not contain certain pneumococcal serotypes that are contained in previously recommended pneumococcal vaccines, one of which is pneumococcal serotype 4.
- In certain adult populations in the western United States, high percentages (i.e., ≥30%) of IPD caused by serotype 4 have occurred. The available IPD serotype data from CDC's Active Bacterial Core surveillance, as well as similar surveillance from Alaska and Navajo Nation, indicate that this serotype is particularly prevalent in Alaska, Colorado, Navajo Nation, New Mexico, and Oregon. Serotype 4 IPD occurs across age groups; however, cases are frequently observed among adults aged <65 years who have underlying conditions, such as alcoholism, chronic lung disease, cigarette smoking, homelessness, and injection drug use. In such populations in these geographic areas, other recommended pneumococcal vaccines (e.g., PCV20 alone or both PCV15 and PPSV23) are expected to provide broader serotype coverage against locally circulating strains compared with PCV21.
- The percentages of serotype 4 IPD cases in other areas of the western United States without IPD surveillance are currently unknown. IPD surveillance from other geographic areas in the United States (e.g., midwestern, eastern, and southern regions) has not detected significant percentages of serotype 4.
- This clinical guidance will be reviewed and updated as pneumococcal disease epidemiology evolves.

**Abbreviations:** IPD = invasive pneumococcal disease; PCV = pneumococcal conjugate vaccine; PCV13 = 13-valent PCV; PCV15 = 15-valent PCV; PCV20 = 20-valent PCV; PCV21 = 21-valent PCV; PPSV23 = 23-valent pneumococcal polysaccharide vaccine.

### PPSV23 Use in PCV13-Experienced Adults Who Have Not Completed the Recommended Vaccination Series

Among adults aged ≥19 years who have started their pneumococcal vaccination series with PCV13 but have not received all recommended doses, PPSV23 is no longer recommended as an option to complete the series. Either PCV20 or PCV21 is recommended to complete the series as previously recommended. (Table).

### Coadministration with Other Vaccines

In accordance with CDC’s General Best Practice Guidelines for Immunization, routine administration of a pneumococcal vaccine with other age-appropriate doses of vaccines at the same visit is recommended for adults who have no specific contraindications to vaccination at the time of the health care visit (*33*).

### Contraindications and Precautions

Vaccination providers should consult the vaccine package insert for precautions, warnings, and contraindications (*4**–**7*). Vaccination with PCV or PPSV23 is contraindicated in persons known to have had a severe allergic reaction (e.g., anaphylaxis) to any component of the vaccine. Because PCVs are conjugated to CRM197, a nontoxic genetically altered diphtheria toxin, these vaccines are also contraindicated in persons known to have had a severe allergic reaction to any diphtheria toxoid–containing vaccine (*4**–**7*).

### Reporting of Vaccine Adverse Events

Adverse events occurring after administration of any vaccine should be reported to VAERS. Instructions for reporting to VAERS are available at https://vaers.hhs.gov/reportevent.html or by calling 800-822-7967.

### Future Research and Monitoring Priorities

CDC and ACIP will continue to assess safety and public health impact of pneumococcal vaccines among adults. This includes monitoring the duration of vaccine-conferred immunity from PCV to determine the need for a booster to ensure that older adults continue to be protected under the updated vaccine recommendation and to measure any indirect effects on incidence in adults from routine childhood vaccination.

## Footnotes

* Alcoholism; cerebrospinal fluid leak; chronic heart, liver, or lung disease; chronic renal failure; cigarette smoking; cochlear implant; congenital or acquired asplenia; diabetes mellitus; generalized malignancy; HIV; Hodgkin disease; immunodeficiency; iatrogenic immunosuppression; leukemia, lymphoma, or multiple myeloma; nephrotic syndrome; solid organ transplant; or sickle cell disease or other hemoglobinopathies.

† https://www.cdc.gov/acip/evidence-to-recommendations/adults-50-64-without-pneumococcal-vaccine-etr.html

§ Defined as a pneumococcal infection in a normally sterile site (e.g., blood, cerebrospinal fluid, bone, or joint space).

¶ PCV21 received indication for protection against IPD serotype 15B based on immunogenicity data. The percentage increases to 85% if serotype 15B is included as part of PCV21 serotype.

** At least one of the following conditions, according to the 2020 National Health Interview Survey: chronic heart disease, chronic lung disease, chronic liver disease, diabetes, smoking, alcoholism, weakened immune system due to prescriptions, weakened immune system due to health condition, solid cancer (not including nonmelanoma skin cancer or unknown type of skin cancer), and blood cancer. The percentages were 32% for non-Hispanic Asian (Asian) adults; 43% for Hispanic or Latino (Hispanic) adults; 50% for non-Hispanic White (White) adults; and 54% for Black adults.

†† According to 2022 Behavioral Risk Factor Surveillance System data, coverage with any pneumococcal vaccine among adults aged 50–64 years with risk-based recommendation by race and ethnicity was 27.9% (Hispanic), 39.3% (White), 38.2% (Black), 36.5% (Asian), and 35.1% (AI/AN); coverage among adults aged ≥65 years by race and ethnicity was 55.1% (Hispanic), 72.7% (White), 63.1% (Black), 64.1% (Asian), and 62.1% (AI/AN).

§§ These cases were based on claims without chart confirmation. Therefore, in addition to the GBS definition used for the primary analysis (*International Classification of Diseases, Tenth Revision, Clinical Modification* [ICD-10-CM] code: G61.0), an alternative definition based on literature search (ICD-10-CM codes: G61.0, G61.81, G61.1, G61.8, and G61.9) was used for sensitivity analysis.

¶¶ The 30% threshold was guided by economic models that showed that once the percentage of cases of pneumococcal disease caused by serotype 4 exceeds 30%, PCV21 use might result in higher cost and, in some cases, worse health outcomes compared with PCV20 use. https://www.cdc.gov/acip/downloads/slides-2024-06-26-28/02-Pneumococcal-Stoecker-508.pdf

## References

[1] CDC. Active bacterial core surveillance (ABCs): ABCs bact facts interactive data dashboard. Atlanta, GA: US Department of Health and Human Services, CDC; 2024. https://www.cdc.gov/abcs/bact-facts/data-dashboard.html

[2] Matanock A, Lee G, Gierke R, Kobayashi M, Leidner A, Pilishvili T. Use of 13-valent pneumococcal conjugate vaccine and 23-valent pneumococcal polysaccharide vaccine among adults aged ≥65 years: updated recommendations of the Advisory Committee on Immunization Practices. MMWR Morb Mortal Wkly Rep 2019;68:1069–75. 10.15585/mmwr.mm6846a5 31751323 PMC6871896

[3] Kobayashi M. Summary of work group interpretation of EtR and policy options: PCV use in adults aged ≥50 years [Presentation slides]. Presented at the Advisory Committee on Immunization Practices meeting, Atlanta, GA; October 23, 2024. https://www.cdc.gov/acip/downloads/slides-2024-10-23-24/04-Kobayashi-Pneumococcal-508.pdf

[4] Food and Drug Administration. Package insert: PREVNAR 20. Silver Spring, MD: US Department of Health and Human Services, Food and Drug Administration; 2024. https://www.fda.gov/media/149987/download?attachment

[5] Food and Drug Administration. Package insert: CAPVAXIVE. Silver Spring, MD: US Department of Health and Human Services, Food and Drug Administration; 2024. https://www.fda.gov/media/179426/download?attachment

[6] Food and Drug Administration. Package insert: VAXNEUVANCE. Silver Spring, MD: US Department of Health and Human Services, Food and Drug Administration; 2024. https://www.fda.gov/media/150819/download?attachment

[7] Food and Drug Administration. Package insert: PNEUMOVAX23. Silver Spring, MD: US Department of Health and Human Services, Food and Drug Administration; 2024. https://www.fda.gov/media/80547/download

[8] Kobayashi M, Leidner AJ, Gierke R, Use of 21-valent pneumococcal conjugate vaccine among U.S. adults: recommendations of the Advisory Committee on Immunization Practices—United States, 2024. MMWR Morb Mortal Wkly Rep 2024;73:793–8. 10.15585/mmwr.mm7336a2 39264843 PMC11392227

[9] Food and Drug Administration. Package insert: Prevnar 13. Silver Spring, MD: US Department of Health and Human Services, Food and Drug Administration; 2024. https://www.fda.gov/files/vaccines%2C%20blood%20%26%20biologics/published/Package-Insert------Prevnar-13.pdf

[10] Leidner AJ, Bletnitsky S. Summary of three economic analyses on the use of PCVs among 50–64 year old adults in the United States [Presentation slides]. Presented at the Advisory Committee on Immunization Practices meeting, Atlanta, GA; October 23, 2024. https://www.cdc.gov/acip/downloads/slides-2024-10-23-24/03-Leidner-Pneumococcal-508.pdf

[11] Kobayashi M, Farrar JL, Gierke R, Use of 15-valent pneumococcal conjugate vaccine and 20-valent pneumococcal conjugate vaccine among U.S. adults: updated recommendations of the Advisory Committee on Immunization Practices—United States, 2022. MMWR Morb Mortal Wkly Rep 2022;71:109–17. 10.15585/mmwr.mm7104a1 35085226 PMC9351524

[12] Kobayashi M, Pilishvili T, Farrar JL, Pneumococcal vaccine for adults aged ≥19 years: recommendations of the Advisory Committee on Immunization Practices, United States, 2023. MMWR Recomm Rep 2023;72:1–39. 10.15585/mmwr.rr7203a1 37669242 PMC10495181

[13] Isturiz R, Grant L, Gray S, Expanded analysis of 20 pneumococcal serotypes associated with radiographically confirmed community-acquired pneumonia in hospitalized US adults. Clin Infect Dis 2021;73:1216–22. 10.1093/cid/ciab375 33982098 PMC8492118

[14] Self WH, Johnson KD, Resser JJ, ; PNEUMO Study Investigators. Prevalence, clinical severity, and serotype distribution of pneumococcal pneumonia among adults hospitalized with community-acquired pneumonia in Tennessee and Georgia, 2018–2022. Clin Infect Dis 2024;79:838–47. 10.1093/cid/ciae316 39016606 PMC11478805

[15] Ramirez J, Furmanek S, Chandler TR, ; The University of Louisville Pneumonia Study Group. Epidemiology of pneumococcal pneumonia in Louisville, Kentucky, and its estimated burden of disease in the United States. Microorganisms 2023;11:2813 10.3390/microorganisms11112813 38004825 PMC10673027

[16] Kobayashi M. Evidence to recommendations framework: PCV20 use among adults who previously received PCV13 [Presentation slides]. Presented at the Advisory Committee on Immunization Practices meeting, Atlanta, GA; October 19, 2022. https://stacks.cdc.gov/view/cdc/122357

[17] Kobayashi M. Summary of work group interpretations of EtR and policy option on PCV21 use in adults [Presentation slides]. Presented at the Advisory Committee on Immunization Practices meeting, Atlanta, GA; June 27, 2024. https://www.cdc.gov/acip/downloads/slides-2024-06-26-28/04-Pneumococcal-Kobayashi-508.pdf

[18] Song JY, Chang CJ, Andrews C, ; V114-016 (PNEU-PATH) study group. Safety, tolerability, and immunogenicity of V114, a 15-valent pneumococcal conjugate vaccine, followed by sequential PPSV23 vaccination in healthy adults aged ≥50 years: a randomized phase III trial (PNEU-PATH). Vaccine 2021;39:6422–36. 10.1016/j.vaccine.2021.08.038 34489128

[19] Mohapi L, Pinedo Y, Osiyemi O, ; V114-018 (PNEU-WAY) study group. Safety and immunogenicity of V114, a 15-valent pneumococcal conjugate vaccine, in adults living with HIV. AIDS 2022;36:373–82. 10.1097/QAD.0000000000003126 34750291 PMC8815827

[20] Platt HL, Cardona JF, Haranaka M, A phase 3 trial of safety, tolerability, and immunogenicity of V114, 15-valent pneumococcal conjugate vaccine, compared with 13-valent pneumococcal conjugate vaccine in adults 50 years of age and older (PNEU-AGE). Vaccine 2022;40:162–72. 10.1016/j.vaccine.2021.08.049 34507861

[21] Severance R, Schwartz H, Dagan R, Safety, tolerability, and immunogenicity of V114, a 15-valent pneumococcal conjugate vaccine, administered concomitantly with influenza vaccine in healthy adults aged ≥50 years: a randomized phase 3 trial (PNEU-FLU). Hum Vaccin Immunother 2022;18:1–14. 10.1080/21645515.2021.1976581 34726574 PMC8920144

[22] Merck Sharp & Dohme. Safety, tolerability, and immunogenicity of V110 or V114 co-administered with a booster dose of mRNA-1273 in healthy adults (V110–911). Rahway, NJ: Merck Sharp & Dohme; 2024. https://ClinicalTrials.gov/show/NCT05158140

[23] Simon JK, Staerke NB, Hemming-Harlo M, ; V114-020 PNEU-TRUE study group. Lot-to-lot consistency, safety, tolerability, and immunogenicity of V114, a 15-valent pneumococcal conjugate vaccine, in healthy adults aged ≥50 years: a randomized phase 3 trial (PNEU-TRUE). Vaccine 2022;40:1342–51. 10.1016/j.vaccine.2021.12.067 35039194

[24] Essink B, Sabharwal C, Cannon K, Pivotal phase 3 randomized clinical trial of the safety, tolerability, and immunogenicity of 20-valent pneumococcal conjugate vaccine in adults aged ≥18 years. Clin Infect Dis 2022;75:390–8. 10.1093/cid/ciab990 34940806 PMC9427137

[25] Hurley D, Griffin C, Young M Jr, Safety, tolerability, and immunogenicity of a 20-valent pneumococcal conjugate vaccine (PCV20) in adults 60 to 64 years of age. Clin Infect Dis 2021;73:e1489–97. 10.1093/cid/ciaa1045 32716500 PMC8492133

[26] Haranaka M, Young Song J, Huang KC, A phase 3 randomized trial of the safety and immunogenicity of 20-valent pneumococcal conjugate vaccine in adults ≥60 years of age in Japan, South Korea, and Taiwan. Vaccine 2024;42:1071–7. 10.1016/j.vaccine.2024.01.004 38267330

[27] Platt H, Omole T, Cardona J, Safety, tolerability, and immunogenicity of a 21-valent pneumococcal conjugate vaccine, V116, in healthy adults: phase 1/2, randomised, double-blind, active comparator-controlled, multicentre, US-based trial. Lancet Infect Dis 2023;23:233–46. 10.1016/S1473-3099(22)00526-6 36116461

[28] Platt HL, Bruno C, Buntinx E, ; STRIDE-3 Study Group. Safety, tolerability, and immunogenicity of an adult pneumococcal conjugate vaccine, V116 (STRIDE-3): a randomised, double-blind, active comparator controlled, international phase 3 trial. Lancet Infect Dis 2024;24:1141–50. 10.1016/S1473-3099(24)00344-X 38964361

[29] Scott P, Haranaka M, Choi JH, ; STRIDE-6 study group. A phase 3 clinical study to evaluate the safety, tolerability, and immunogenicity of V116 in pneumococcal vaccine-experienced adults 50 years of age or older (stride-6). Clin Infect Dis 2024;79:1366–74. 10.1093/cid/ciae383 39082735 PMC11650886

[30] Merck Sharp & Dohme. A phase 3 randomized, double-blind, placebo-controlled clinical study to evaluate the safety, tolerability, and immunogenicity of V116 when administered concomitantly with influenza vaccine in adults 50 years of age or older. Rahway, NJ: Merck Sharp & Dohme; 2023. https://clinicaltrials.gov/study/NCT05526716

[31] Merck Sharp & Dohme. A phase 3, multicenter, randomized, double-blind, active comparator-controlled study to evaluate the safety, tolerability, and immunogenicity of V116 in adults living with HIV. Rahway, NJ: Merck Sharp & Dohme; 2023. https://clinicaltrials.gov/study/NCT05393037

[32] Merck Sharp & Dohme. Safety and immunogenicity of V116 in pneumococcal vaccine-naïve adults 50 years of age or older (V116–010, STRIDE-10). Rahway, NJ: Merck Sharp & Dohme; 2024. https://www.clinicaltrials.gov/study/NCT05569954?term=V116-010&rank=1

[33] Kroger A, Bahta L, Long S, Sanchez P. General best practice guidelines for immunization. Atlanta, GA: US Department of Health and Human Services, CDC; 2024. www.cdc.gov/vaccines/hcp/acip-recs/general- recs/downloads/general-recs.pdf

